# Helarchaeota and co-occurring sulfate-reducing bacteria in subseafloor sediments from the Costa Rica Margin

**DOI:** 10.1038/s43705-021-00027-x

**Published:** 2021-06-17

**Authors:** Rui Zhao, Jennifer F. Biddle

**Affiliations:** School of Marine Science and Policy, University of Delaware, Lewes, DE USA

**Keywords:** Water microbiology, Microbial ecology

## Abstract

Deep sediments host many archaeal lineages, including the Asgard superphylum which contains lineages predicted to require syntrophic partnerships. Our knowledge about sedimentary archaeal diversity and their metabolic pathways and syntrophic partners is still very limited. We present here new genomes of Helarchaeota and the co-occurring sulfate-reducing bacteria (SRB) recovered from organic-rich sediments off Costa Rica Margin. Phylogenetic analyses revealed three new metagenome-assembled genomes (MAGs) affiliating with Helarchaeota, each of which has three variants of the methyl-CoM reductase-like (MCR-like) complex that may enable them to oxidize short-chain alkanes anaerobically. These Helarchaeota have no multi-heme cytochromes but have Group 3b and Group 3c [NiFe] hydrogenases, and formate dehydrogenase, and therefore have the capacity to transfer the reducing equivalents (in the forms of hydrogen and formate) generated from alkane oxidation to external partners. We also recovered five MAGs of SRB affiliated with the class of Desulfobacteria, two of which showed relative abundances (represented by genome coverages) positively correlated with those of the three Helarchaeota. Genome analysis suggested that these SRB bacteria have the capacity of H_2_ and formate utilization and could facilitate electron transfers from other organisms by means of these reduced substances. Their co-occurrence and metabolic features suggest that Helarchaeota may metabolize synergistically with some SRB, and together exert an important influence on the carbon cycle by mitigating the hydrocarbon emission from sediments to the overlying ocean.

## Introduction

Many of the total microbial cells in the marine environment are estimated to be present in marine sediments [1], of which a considerable fraction are archaea [2–4]. Although archaeal communities in oligotrophic and often oxic sediments are dominated by aerobic ammonia-oxidizing Thaumarchaeota [5–8], those in coastal and often organic-rich sediments are more diverse and complex [9], and their metabolic activity exerts a critical influence on the carbon and nutrient cycling on the global scale (e.g., [10–12]).

The Asgard superphylum of archaea is of evolutionary importance because its present-day members are thought to share a common ancestor with modern eukaryotes [13, 14]. The archaeal phyla of this superphylum, including but not limited to Lokiarchaeota, Thorarchaeota, Odinarchaeota, and Heimdallarchaeota, have been established by metagenome-assembled genomes (e.g., [13, 15]). A recent analysis of available Asgard 16S rRNA gene sequences [16] showed that some sequences cannot be resolved to the existing lineages and thus suggested the diversity of Asgard archaea is likely broader than currently recognized [17]. Marine sediment is the natural habitat of the only cultured Asgard archaeon *Candidatus* Prometheoarchaeum syntrophicum MK-D1 [18] and the vast majority of known Asgard archaea discovered via metagenomic analysis [13, 14], so further exploration of marine sediment is promising to increase our understanding about this fascinating branch of life by recovering more novel lineages. This speculation is supported by the recent discovery of new Asgard lineages, such as Helarchaeota [19] in hydrothermal sediments in the Guaymas Basin (GB) and Sifarchaetoa in organic-rich sediments of the Costa Rica Margin [20].

Genome analysis and laboratory cultures suggest that some Asgard archaea in marine sediments will need external partner cells to consume the reducing equivalents (e.g., in the forms of hydrogen and formate) generated during organic matter degradation [18, 21]. Helarchaeota from GB encode the Methyl-CoM reductase-like complex (MCR-like complex) and are proposed to be capable of oxidizing short-chain alkanes to conserve energy [19], using a pathway similar as other thermophilic alkane-oxidizing Euryarchaeota enriched from the same location [22, 23]. Most of these characterized alkane-oxidizing archaea form consortia with sulfate-reducing bacteria (SRB) that use the reducing equivalents released during alkane oxidation for sulfate reduction [22–24], although some alkane-oxidizing archaea can also channel the electrons to their own internal methanogenesis pathway [24, 25]. As a prevalent SRB in hydrothermal sediments, *Candidatus* Desulfofervidus auxilii [26] is the dominant partner bacteria of the characterized alkane-oxidizing Euryarchaeota in heated marine sediments [22, 23]. However, the potential sinks of the reducing equivalents of Helarchaeota in other sediment environments remain elusive.

Scientific drilling off the Costa Rica Margin during International Ocean Drilling Program (IODP) Expedition 334 provided an excellent avenue to explore the diversity of archaea, because sediments from this location were shown to harbor high abundances [27] and also phylogenetically novel lineages of archaea [20, 28]. The drilled sites are in the forearc basin of the subduction zone, where a considerable fraction of carbon exchange between Earth’s surface and interior occurs [29]. Abundant thermogenic alkanes (ethane, propane, and butane) were detected in the deep sediments, but not the surface sediments [30, 31]. This prompted us to perform further metagenomic sequencing and analysis on shallow sediment samples from this expedition, to explore the mechanisms of alkane depletion in this unique setting. Here, we report three new Helarchaeota metagenome-assembled genomes (MAGs) in shallow sediments of Hole U1379B, each of which has three variants of the MCR-like complex and could be significant alkane-oxidizing archaea in this sediment. These Helarchaeota account for the majority of the mcrA-bearing archaea community, with the rest of *mcrA*-bearing archaea affiliated to the typically methanotrophic family of ANME-1. We also recovered five novel MAGs of sulfate-reducing bacteria in the class of Desulfobacteria, some of which could be the syntrophic partners of Helarchaeota due to their co-occurring pattern and their matched metabolic capacities of H_2_ and formate utilization. This study expands our understanding about the diversity, metabolic functions, and interspecies dependency of Helarchaeota, which could exert a profound influence on the volatile carbon exchange between marine sediments and the overlying seawater.

## Results and discussion

### Geochemical context

**Fig. 1:**
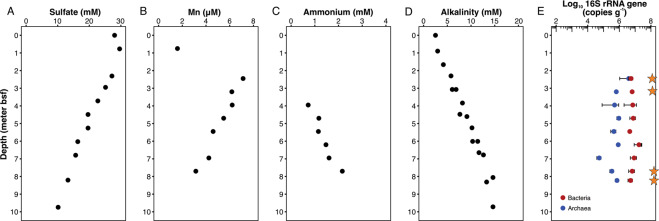
Porewater profiles and microbial abundances in the sediments at IODP Site 1379 on the Costa Rica margin. Porewater profiles in meters below seafloor (meters bsf) were compiled from previous reports/publications of IODP Expedition 334 [30, 52]. Porewater profiles of sulfate (**A**), dissolved manganese (**B**), ammonium (**C**), and alkalinity (**D**) in the upper 10 m sediments in Hole U1379. **E** Abundances of archaeal and bacterial 16S rRNA genes quantified using qPCR with domain-specific primers. Stars denote the four sediment horizons where metagenome sequencing data was generated for this study.

IODP Site U1379 is located on the upper slope of the erosive subduction zone off the Osa Peninsula, Costa Rica. The sediment pile at this site is estimated to be 890 m thick and is characterized by high sedimentation rates of 1.60–10.35 cm/ky [30] and low porosities of ~0.6 [30]. We focused this study on shallow sediments, 2–9 m below seafloor (mbsf), of U1379B, including a total of eight subsampled whole-round cores. Sediments in this depth interval are within the sulfate reduction zone with porewater sulfate concentration decreasing with depth (Fig. 1A). The sulfate-depletion depth was not examined in this study but was noted at ~30 mbsf [30]. Dissolved Mn concentrations in the porewater show the same decreasing trend as sulfate (Fig. 1B), indicating that Mn^2+^ is mainly consumed rather than produced, and therefore Mn oxides might not be the major terminal electron acceptor. The increasing porewater concentrations of ammonium (Fig. 1C) and alkalinity (Fig. 1D) with depth reflect the ongoing degradation of organic matter, with sulfate as the most prominent electron acceptor based on available measurements.

### Microbial abundances and overall community structures

We used domain-specific primers [6] to quantify the abundance of archaeal and bacterial 16S rRNA genes in the nine sediment horizons from 2 to 9 mbsf of Hole U1379B. Both archaeal and bacterial 16S rRNA gene abundances are stable throughout the examined sediments (Fig. 1E). However, archaea are about one order of magnitude lower than bacteria, resulting in bacterial dominance in most of the studied samples except the shallowest sample (~2 mbsf).

**Fig. 2:**
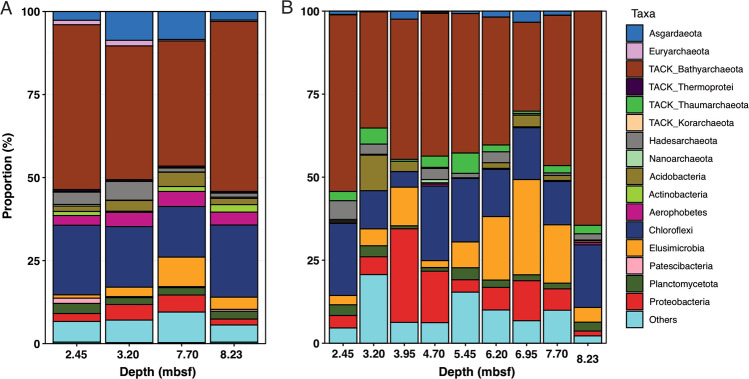
Microbial community structures assessed by 16S rRNA gene reads in raw metagenome sequencing data (**A**) and amplicon sequencing (**B**). For **A**, unassembled reads in the metagenomes were mapped to the SILVA 132 release and classified using phyloFlash. In both **A** and **B**, taxa <5% relative abundance was grouped into “Others”.

In order to maximize archaeal genome recovery, we selected four sediment horizons (2.45, 3.20, 7.70, and 8.23 mbsf) for shotgun metagenome sequencing, due to the high proportions of archaea in these sediment horizons assessed by qPCR (Fig. 1E). We examined the microbial community structures in these four samples by 16S rRNA gene analyses based on both the un-assembled metagenome reads and the 16S rRNA gene amplicon sequencing data (Fig. 2). Based on the metagenome data, in the archaeal domain, Bathyarchaeota is the major archaeal phylum and accounts for on average 44.7% (37.7–51.2% in individual samples) of the total communities, while Asgard archaea especially Lokiarchaeota were also detected to account for on average 5.6% (2.6–8.7% in individual samples) of the total communities (Fig. 2a). In the bacterial domain, Chloroflexi is the most dominant phylum, which accounts for on average 19.0% of the total communities in different depths. Other major bacterial phyla include Acidobacteria, Actinobacteria, Elusimicrobia, Planctomycetes, and Proteobacteria (mainly Deltaproteobacteria) (Fig. 2A). Similar results were also obtained from the 16S rRNA gene amplicon sequencing and analysis (Fig. 2B). These results are consistent with the previous assessments of microbial community structures of marine sediments off Costa Rica [27, 28].

### The presence and diversity of mcrA-bearing microbes

As common products of thermocatalytic degradation of organic matter in subduction zones of high temperature and pressure, C_2_–C_5_ alkanes (ethane, propane, butane, and pentane) were measured in sediments deeper than 50 mbsf [30] (Fig. S1). Alkanes in U1379 were generally not detected in the sediments shallower than 50 mbsf and no flux toward the overlying water column can be quantified, suggesting that most of the upward diffusing fluxes of alkanes are consumed in the shallow sediments. To examine the presence and overall diversity of potentially alkane-consuming archaea in the CR sediments, we employed the GraftM program [32] to analyze the *mcrA* gene (encoding the methyl-CoM reductase alpha subunit, the key enzyme of methane/alkane metabolism) in the metagenome datasets, in which analysis putative *mcrA* gene sequences were identified using HMMs and classified by placing onto the pre-constructed phylogenetic tree described in [33]. Our results revealed that *mcrA* gene was present in all the four sediment horizons where the metagenomes sequencing data were generated from. Except for 3.20 mbsf, the *mcrA*-containing archaeal communities in all the other three horizons were not dominated by well-known archaea in the Euryarchaeota phylum (Figs. S2 and S3), indicating that under-studied archaea may be the dominant methane/alkane metabolizing microbes in the CR sediments, which supports the recently expanded phylogenetic breadth of methane/alkane-metabolizing archaea [24, 34, 35].

### Helarchaeota MAGs from CR sediments

**Fig. 3:**
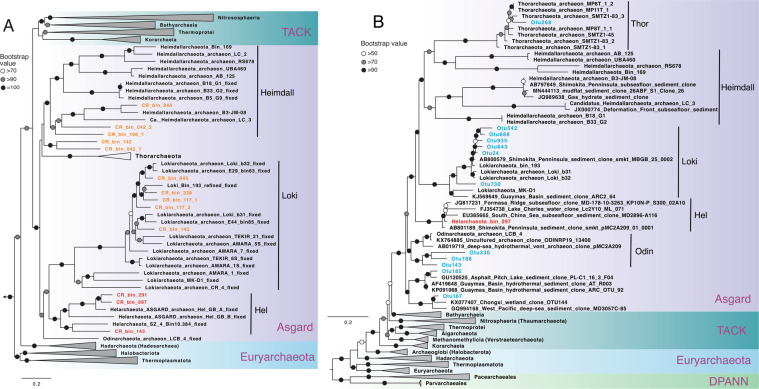
Maximum-likelihood phylogenetic tree of 15 concatenated ribosomal proteins (**A**) and 16S rRNA gene (**B**) of archaea. **A** Phylogenetic tree of 15 concatenated ribosomal proteins inferred using IQ-TREE v1.6.10 with the LG + R7 model and 1000 ultrafast bootstraps. The Helarchaeota MAGs recovered in this study are highlighted in red. Asgard archaea MAGs other than Helarchaeota recovered in this study are shown in orange, phyla are labeled with abbreviated names. **B** Tree of 16S rRNA genes inferred using IQ-TREE v1.6.10 with the SYM + R5 model and 1000 ultrafast bootstraps. The Helarchaeota MAG is highlighted in red, while the short OTUs (287 bp) affiliated with Asgard archaea are shown in blue. Lineages were collapsed at the phylum level, except for those in the Asgard superphylum. The scale bars show estimated sequence substitutions per residue.

Through metagenome assembly and binning, we obtained 12 archaeal MAGs that could be taxonomically assigned to the Asgard superphylum, three of which are affiliated with the newly proposed Helarchaeota phylum [19], four Lokiarchaeota, and three Heimdallarchaeota (Fig. 3A). These three Helarchaeota MAGs (CR_Bin_097, CR_ Bin_143, and CR_ Bin_291) expand the diversity of this newly proposed phylum, doubling the number of available Helarchaeota genomes [19, 36]. The genome sizes of the three CR Helarchaeota MAGs are similar to SZ_4_Bin10.384 (4.7 Mbp) recovered from coastal sediments [36], but are larger than two Helarchaeota genomes recovered from GB (4.7–5.4 Mbp versus 3.54–3.84 Mbp for GB Helarchaeota; Table 1). The CR Helarchaeota MAGs have 4592–4957 coding sequences distributed on 212–589 scaffolds (Table 1). Based on the calculated average amino acid identity (AAI), the six available Helarchaeota genomes could be resolved to four genera (i.e., with <65% intra-genera AAIs; [37]): Hel_GB_A, CR_Bin_097, and CR_Bin_291 belong to a genus, while each of the other three genomes represents a separated genus (Fig. S4).

**Table 1:** Summary of the Helarchaeota and sulfate-reducing bacteria MAGs recovered from Hole U1379B.

Group	MAG ID	Genome size (Mbp)	# Scaffolds	16S rRNA gene (bp)	% GC	Completeness	Redundancy	Strain heterogeneity	N50 of contigs (bp)	# Coding sequences	Coding density (%)	rRNAs	tRNAs	MHCs
Helarchaeota	Helarchaeota Bin_097	5.4	212	1191	38.60	91.60%	3.30%	0	51,451	4957	89.7	1	11	0
	Helarchaeota Bin_291	4.7	475	–	36.40	83.20%	5.60%	0	16,908	4592	88.7	1	13	0
	Helarchaeota Bin_143	4.7	589	–	30.70	92.20%	4.70%	25%	13,188	4805	87.5	0	25	0
ANME-1 archaea	CR_Bin_179	2.5	136	1483	41.90	90.30%	2.60%	20%	29,614	2675	88.5	3	42	10
Sulfate-Reducing Bacteria	CR_Bin_276	4.3	591	743	49.50	92.30%	3.40%	0	13,703	4605	84.7	1	43	15
	CR_Bin_073	2.2	197	–	48.20	90.20%	0	0	19,399	2132	87.5	0	46	24
	CR_Bin_129	3	509	–	48.00	77.60%	3.40%	16.70%	9247	3131	85.4	0	32	4
	CR_Bin_060	2.2	532	–	49.20	71.20%	3.20%	20.00%	5293	2441	87.6	0	30	5
	CR_Bin_277	1.5	495	–	51.50	64.60%	1.90%	33.30%	3516	1,685	86.6	0	15	1

Among the three CR Helarchaeota MAGs, only CR_Bin_097 has a reconstructed partial (1191 bp) 16S rRNA gene sequence. This is notable since none of the previously reported Helarchaeota MAGs has a 16S rRNA gene sequence [19, 36]. An alignment of the 16S rRNA gene sequence of Helarchaeota CR_Bin_097 and other archaea revealed three insertions of >20 bp in this sequence and also other environmental clones of Asgard archaea. These insertions unfortunately prevent a perfect match between Helarchaeota CR_Bin_097 and the amplicon operational taxonomy units (OTUs), making it challenging to accurately estimate the distribution of these Helarchaeota in the shallow sediment horizons of Hole U1379. However, based on the read recruiting and genome coverage calculation, the three Helarchaeota seem to have similar distribution patterns and be highest in the depth of 7.70 mbsf sample (Fig. S5). The 16S rRNA gene of CR_Bin_097, after manual removal of the insertions, showed nucleotide identities of >90% to diverse uncultured archaeal sequences from marine anoxic sediments, which together formed a monophyletic clade in the phylogenetic tree of archaeal 16S rRNA gene (Fig. 3B), supporting that Helarchaeota is a separate phylum within the Asgard superphylum.

### Alkane oxidation potential of CR Archaea

**Fig. 4:**
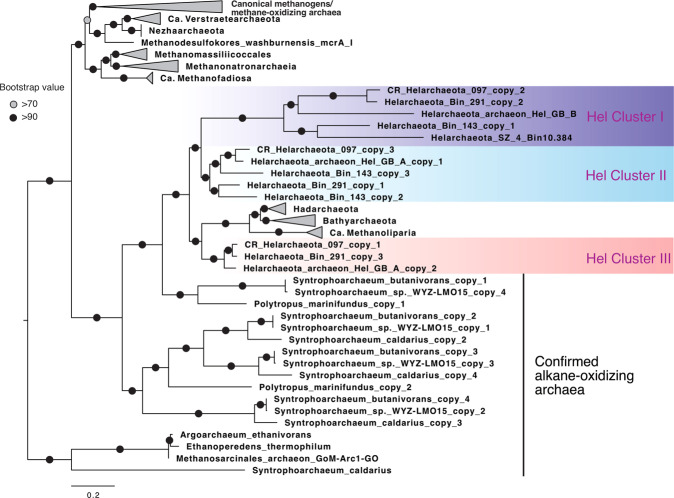
Maximum-likelihood phylogeny of methyl-coenzyme M reductase submit alpha (McrA) of archaea. This tree was inferred using IQ-TREE v1.6.10 with LG + F + I + G4 as the best-fit evolutionary model and 1000 ultrafast bootstraps based on an alignment of 450 amino acid positions of 167 sequences from diverse MCR-containing archaea. The McrA sequences of Helarchaeota were placed into three distinct clusters: Hel Cluster I, II, and III. The scale bar shows estimated sequence substitutions per residue.

Similar to the GB Helarchaeota [19], Helarchaeota in CR sediments have the potential of alkane oxidation. Each of the three CR Helarchaeota MAGs has three variants of methyl-CoM reductase-like enzymes (McrABG operon, or MCR) (Fig. 4), whereas the previously reported Helarchaeota MAGs [19, 36] have only 1–2 MCR variants. Phylogenetic analyses of the alpha subunit of methyl-CoM reductase-like enzymes (McrA) revealed that McrA sequences of the six available Helarchaeota MAGs form three clusters (Hel Clusters I, II, and III) within a monophyletic cluster together with homologs of Hadesarchaea, Bathyarchaeota, and *Ca*. Methanoliparia (Fig. 4). These lineages also form a monophyletic clade with McrA homologs of Syntrophoarchaea, *Argoarchaeum ethanivorans*, and *Ethanoperedens thermophilum* [38], which are all alkane-oxidizing Euryarchaeota confirmed by laboratory incubations [22–24]. Because *mcr*-like genes so far have not been found in any Asgard phylum other than Helarchaeota, *mcr* genes in Helarchaeota, as recently suggested [35], may not be vertical inherited from the ancestor of Asgard archaea but instead may have resulted from horizontal gene transfer events from alkane-oxidizing Euryarchaeota although the timing is still uncertain. The three Hel clusters of McrA are divergent, with intra-cluster similarities in the range of 44–63%. Multiple divergent *mcr*-like genes were also reported in “Ca. Syntrophoarchaeum” genomes, in which the duplicated *mcr*-like genes have evolved to use substrates other than methane, such as butane and propane [22]. These CR Helarchaeota MAGs also possess genes associated with the archaeal Wood-Ljungdahl, fatty acids beta-oxidation, and other pathways similar to those found in the characterized alkane-oxidizing genomes [22, 38] that have been recently identified [19]. Therefore, the gene duplication in Helarchaeota may lead to the neofunctionalization [39] of the MCR-like complex, and enable them to metabolize short-chain alkanes of different lengths anaerobically.

Among the MCR-containing MAGs from CR sediments, in addition to the three Helarchaeota MAGs, we also recovered an MAG (CR_Bin_179) affiliated with ANME-1. Based on the phylogenetic analysis of the 15 concatenated ribosomal proteins [40], this MAG should represent a new genus within the family of ANME-1 (Fig. S6A), which is also supported by the classification of GTDB-tk [41] using the 122 single-copy genes of archaea, and the calculated 69%-76% AAI between this MAG and the others in the family of ANME-1 (Supplementary Table S1). CR_ANME1_Bin_179 has a near full-length 16S rRNA gene sequence (1487 bp) (Fig. S6B), which shows an identity of 94.9% with that of ANME1_CONS3730B06UFb1 (a MAG recovered from Hydrate Ridge methane seep sediments [42]) and even lower identities with the other genomes in the family of ANME-1, supporting that this genome should represent a new genus [37]. Its genomic close relatives include ANME1_CONS3730B06UFb1 recovered from Hydrate Ridge methane seep sediments, M5.MMPM from Aarhus Bay sediments, and ANME-1-THS recovered from Tibetan hot spring sediments, for all of which methane metabolism pathways have been proposed [34, 42, 43]. CR_ANME1_Bin_179 may be involved in methane metabolisms, which was supported by the partial MCR operon (constituted by McrB and McrG but the McrA is missing) detected in this genome.

To assess the representability of these McrA-bearing genomes in the CR sediments, we performed a phylogenetic analysis of McrA amino acid sequences in the bulk metagenome assemblies of the four CR sediment layers. Our results indicate that all McrA sequences from CR metagenomes were affiliated with either the three Helarchaeota clusters or the ANME-1 cluster (Fig. S3). Because CR_ ANME1_Bin_179 may only be involved in methane but not alkane metabolisms, CR Helarchaeota could be responsible for the majority of alkane depletion in the surface sediments at U1379.

### Hydrogenases in Helarchaeota

**Fig. 5:**
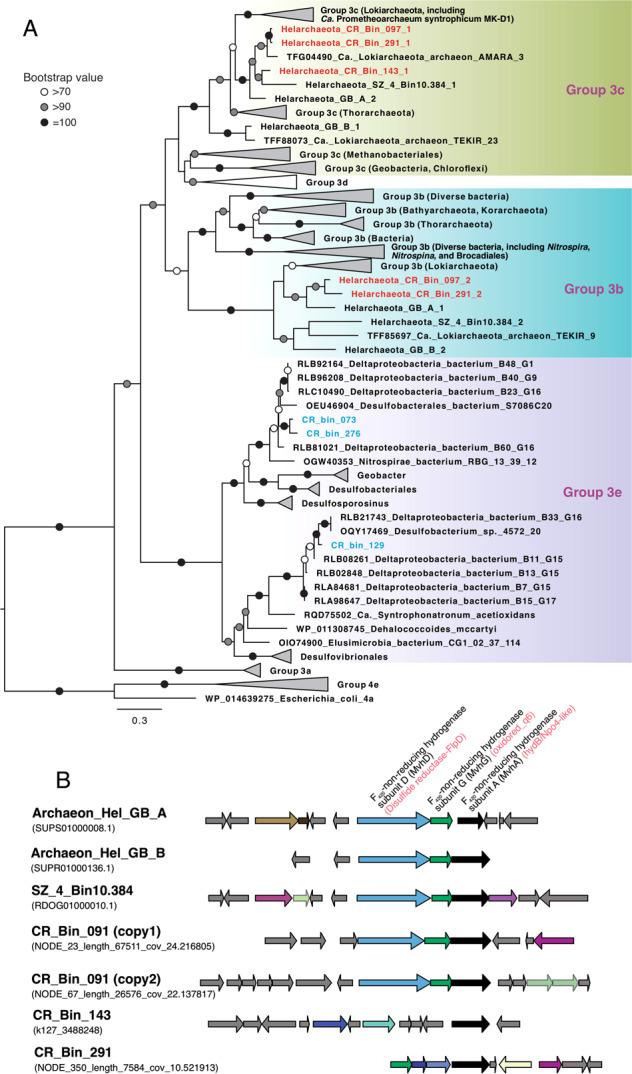
Phylogeny and conservation of Group 3 [NiFe] hydrogenase. **A** Maximum-likelihood phylogenetic tree of Group 3 [NiFe] hydrogenase alpha subunit. This tree was inferred using IQ-TREE v1.6.10 with LG + R6 as the best-fit evolutionary model and 1000 ultrafast bootstraps based on an alignment of 394 positions of protein sequences from diverse Bacteria and Archaea. Sequences of Helarchaeota recovered from CR are shown in red, while those from SRB MAGs are highlighted in blue. The tree is rooted in Group 4 [NiFe] hydrogenase sequences. The scale bar shows estimated sequence substitutions per residue. **B** Synteny of the gene cluster proposed by ref. [19]. as the energy-transfer complex in Helarchaeota. Only annotations of the gene clusters are showed at the top, with annotations from ref. [19]. highlighted in red. Genes shown in gray arrows are hypothetical proteins without annotated function. Genes shown in other colors are annotated with various functions unrelated to energy transfer.

Alkane-oxidizing archaea need to employ various means to channel the reducing equivalents generated during the oxidation of alkanes to either another internal metabolic pathway [25] or to external partner cells [22–24]. Helarchaeota MAGs recovered from CR likely have to depend on bacterial partners as external electron sinks because they do not have (1) the internal pathways for the reduction of sulfate or nitrate/nitrite, (2) multi-heme cytochromes (Table 1), or (3) Type IV electric pilins for extracellular electron transfer to solid electron acceptors (e.g., metal oxides). Instead, these Helarchaeota MAGs from CR contain Group 3 [NiFe] hydrogenases and formate dehydrogenases that could facilitate the transfer of the reducing equivalents in the forms of H_2_ and formate. Phylogenetic analysis of the hydrogenase subunit alpha indicates that all Helarchaeota MAGs have one Group 3b [NiFe] hydrogenase and one Group 3c [NiFe] hydrogenase (Fig. 5A), except for CR_Bin_143 where the lack of recovery may be due to its lower genome completeness. Helarchaeota Group 3b [NiFe] hydrogenases formed a monophyletic cluster with homologs of diverse Lokiarchaeota genomes [21], but were divergent from other Group 3b lineages (Fig. 5A). Similarly, Helarchaeota Group 3c hydrogenases also formed a monophyletic cluster with homologs from diverse Lokiarchaeota and Thorarchaeota genomes [21] (Fig. 5A). Interestingly, the only hydrogenase in the closed genome of the cultured *Ca*. Prometheoarchaeum syntrophicum MK-D1 [18] is also affiliated with this cluster. *Ca*. Prometheoarchaeum syntrophicum was revealed to depend on partnering cells to consume the reduced equivalents in the forms of H_2_ and/or formate [18], in which the Group 3c hydrogenase may be dedicated to this function. It is worth noting that the gene cluster proposed by Seitz et al. [19] as the energy-transfer complex in Helarchaeota contains the Group 3b hydrogenase alpha subunit (hydB/Nqo4-like in ref. [19].). In addition to the two Helarchaeota from GB, this gene cluster is also conserved in the genomes of Helarchaeota CR_Bin_097 (two variants) and Helarchaeota SZ_4_Bin10.384 (Fig. 5B). The congruence between the phylogenies of Asgard Group 3 hydrogenases and their genomes suggested that these hydrogenases may be vertically inherited from their ancestor rather than horizontal transferred from other organisms. Even though these Asgard-specific Group 3 [NiFe] hydrogenases are probably not membrane-bound, they could still play an important role in hydrogen production and transfer between organisms, given that the genome of the cultured Lokiarchaeota has a single Group 3c [NiFe] hydrogenase (Fig. 5A).

### Co-occurrence of sulfate-reducing bacteria and Helarchaeota

**Fig. 6:**
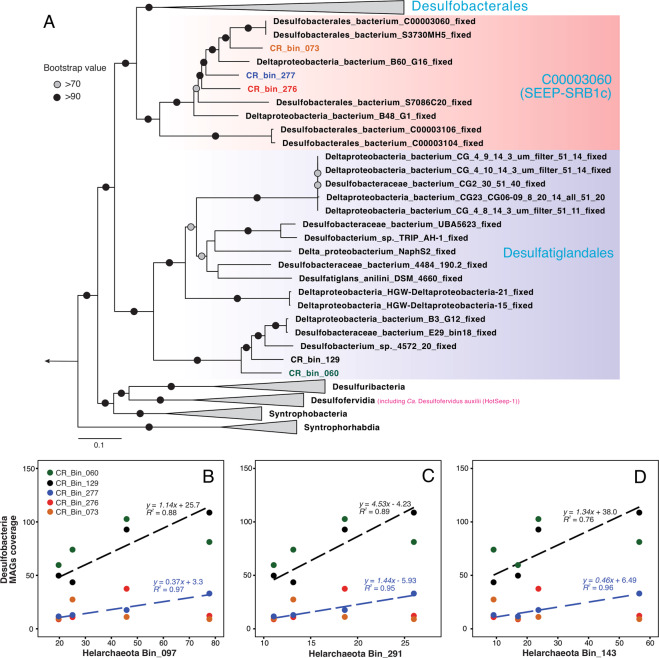
Phylogeny of Desulfobacteria genomes and their genome coverage correlations with Helarchaeota genomes in CR sediments. **A** Maximum-likelihood phylogenetic tree of Desulfobacteria genomes recovered from CR sediments. This tree was inferred using IQ-TREE v1.6.10 with LG + F + R6 as the best-fit evolutionary model and 1000 ultrafast bootstraps, based on an alignment of the concatenated 15 ribosomal proteins sequences. This tree is rooted in genomes of the Desulfurmonadota phylum. Desulfobacteria MAGs recovered from Costa Rica margin sediments are color-coded, while the reference genomes are shown in black. The sulfate-reducing bacterial partner of thermophilic alkane-oxidizing archaea, *Candidatus* Desulfofervidus auxilli in the class of Desulfofevidia, is highlighted in purple. The scale bar shows estimated sequence substitutions per residue. **B–D** Correlations of the genome coverages between Helarchaeota and Desulfobacteria recovered from CR margin sediments. The blue and black dashed lines show the linear correlations of CR_Bin_277 and CR_Bin_129, respectively, with the three Helarchaeota MAGs from CR.

**Fig. 7:**
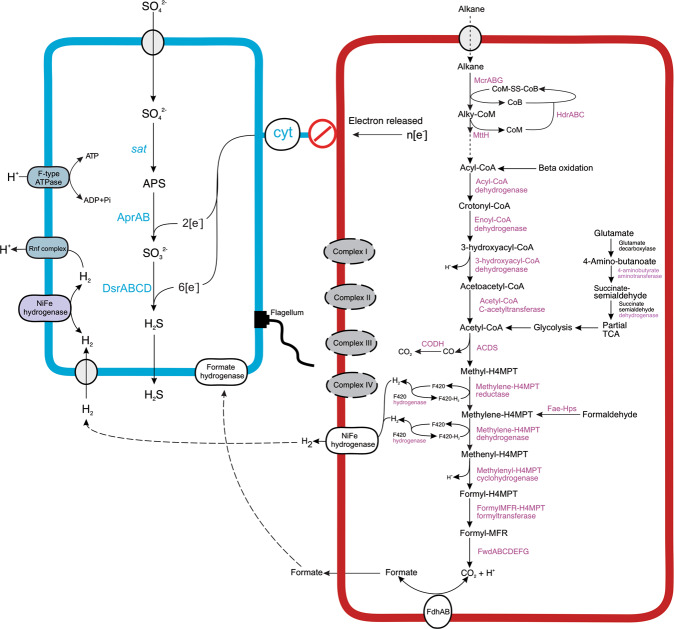
Proposed metabolic scheme and potential interaction between Helarchaeota (red) and sulfate-reducing bacteria (blue) in Costa Rica Margin sediments. Missing genes/pathways in the two genomes and the presumed substrate exchanges between them are shown in dashed lines. Absent membrane complexes are shown in gray. Cyt, (multi-heme) cytochromes; CODH, carbon monoxide dehydrogenase; ACDS, acetyl-CoA decarbonylase/synthase.

The potential partner bacteria of Helarchaeota have not been identified previously, as no genomes were found co-occurring with Helarchaeota MAGs in previous studies [19]. From the same metagenome sequencing dataset, we recovered five bacterial MAGs affiliated to the phylum of Desulfobacterota (Figs. 6A and 7), which was expected due to these samples being taken from the sulfate reduction zone (Fig. 1). Four of them have the complete dissimilatory sulfate reduction pathway (constituted by sulfate adenylyltransferase (sat), adenylyl-sulfate reductase (AprAB), and dissimilatory sulfite reductase (DsrAB)), while the incompletion of this pathway in CR_bin_60 could be due to the low genome completion (71% based on bacterial single-copy genes, Table 1). Phylogenomic analysis based on the 15 concatenated ribosomal proteins suggested that all the five SRB MAGs from Costa Rica margin sediments are affiliated to the class of Desulfobacteria in the phylum Desulfubacterota, according to the genome-based taxonomy classification frame [44], and are different from *Ca*. Desulfofervidus auxilii [26], the partner bacterium of thermophilic alkane-oxidizing archaea, which is affiliated to the class of Desulfofervidia (Fig. 6A). CR_bin_073, CR_bin_276, and CR_bin_277 are members of the order of “C00003060” (Fig. 6A), which corresponds to the lineage of SEEP-SRB1c [42]. The close relative genomes in the order of C00003060 are exclusively recovered from hydrocarbon-rich marine sediments, such as cold seep sites on Hydrate Ridge off the Pacific coast [42], and B60_G16 recovered from GB hydrothermal sediments [45]. The other two MAGs, CR_bin_129 and CR_bin_060, formed a new lineage within the order of Desulfatiglandales, together with MAGs recovered from hydrocarbon-rich sediments in GB [45] and Gulf of Mexico [46] (Fig. 6A).

Among the five SRB MAGs, only CR_Bin_276 has an identifiable partial (743 bp) 16S rRNA gene. It shows >99% identity (with two mismatches) with OTU_8 of the amplicon sequencing data. Due to the difficulties to link the Helarchaeota and SRB MAGs with short amplicon OTUs (i.e., Helarchaeota_Bin_097 does not match with any OTUs, while only one SRB has a 16S rRNA gene and matches with OTU_8), we use genome coverage as a proxy to represent the relative abundance of MAGs in each of the four metagenome samples. We found that all CR Helarchaeota MAG coverages show positive linear correlations with two of the five SRB MAGs (CR_bin_129 and CR_bin_277) (Fig. 6B-6D), representing both lineages of SRB recovered from CR. To attempt to catch these MAGs in action, we also calculated iRep, the index of replication [47], of Helarchaeota and SRB genomes in the CR sediments. iRep of all three Helarchaeota MAGs was only detected in 7.70 mbsf, while among the SRB MAGs only CR_Bin_129 had a calculable iRep (Fig. S7), suggesting CR_Bin_129 is active and co-occurring with Helarchaeota in the CR sediments.

In addition to the abundance correlations, metabolic pathway analysis of the MAGs also support that the co-occurring Helarchaeota and SRB bacteria in CR sediments may engage in synergy metabolism, in which SRB are capable of utilizing the products of the alkane oxidation activity of Helarchaeota, H_2_ and formate (Fig. 7). Three of the five SRB MAGs from CR (CR_bin_073, CR_bin_129, and CR_bin_276) contain a periplasmic hydrogenase affiliated to a novel lineage we describe as Group 3e in the Group 3 [NiFe] hydrogenases (Fig. 5A). This lineage was not well resolved in previous phylogenetic analysis [48] probably due to the small number sequences then available. This hydrogenase may enable these SRBs to conserve energy from the oxidation of H_2_, similar as *D. auxilii*, which contains a periplasmic hydrogenase and is capable of growing using H_2_ in the absence of an ANME partner [26]. However, the bi-directional nature of hydrogenase may enable it to produce hydrogen. Future studies with laboratory cultures are needed to verity this. All the five SRB MAGs additionally have two copies of respiratory formate dehydrogenases, indicating that they also have the capacity of oxidizing formate that is potentially released by the Helarchaeota as a form of reducing equivalents (Fig. 7).

The SBR genomes from CR sediments also contain the full gene set for the synthesis of flagella, which have previously been reported to be an important feature of syntrophy establishment [49] and could be important in initiating the contact between SRB and Helarchaeota.

### Sulfate-dependent alkane oxidation is thermodynamically favorable

To explore if this co-occurrence is energetically favorable, we calculated the Gibbs free energies of the oxidation of short-chain alkanes (ethane, propane, and butane) when coupled to sulfate reduction (Supplementary Table S2) under the near in situ conditions in the shallow sediments at CR. Our results showed that the Gibbs free energy of the sulfate-dependent alkane oxidation (in the unit of kJ m^−1^ alkane) is feasible over a wide range of alkane concentrations (10^−6^–1 µM), and is highest for butane, followed by propane and ethane (Fig. S8). Importantly, these short-chain alkanes can provide more energy than methane per mole of oxidized alkane (Fig. S8), suggesting that these alkanes are more energy-rich than methane and could be more easily consumed by microbes such as the Helarchaeota and SRB detected in CR.

Although more data are required to properly assess the ecological roles of Helarchaeota in the environment, all known Helarchaeota so far are found in hydrocarbon-rich marine sediments [19] and seem to be intrinsically related to the oxidation of alkanes. If they have a sulfate-reducing bacterial partner, their syntrophic metabolic reactions, the sulfate-dependent alkane oxidations, are also high energy-yielding processes. Analogous to the role anaerobic methane-oxidizing archaea have on reducing methane emission from marine sediments [50], the activities of Helarchaeota and SRB in marine sediments may convert alkane to CO_2_ at the expense of sulfate reduction and, therefore, reduce the emission of alkanes into the overlying water column (Fig. S1).

## Conclusion

This study revealed that the majority of Asgard archaeal cells in sulfate-reducing organic-rich shallow sediments co-occur with SRB in the subduction zone off Costa Rica. Among these are three new Helarchaeota identified as MAGs, each of which have three variants of the MCR operon, and may anaerobically oxidize the steadily available alkane that is thermogenically produced from organic matter degradation in deep sediments. Like other characterized thermophilic alkane-oxidizing archaea, these Helarchaeota may engage in a syntrophic relationship with the co-occurring sulfate-reducing bacteria from the class of Desulfobacteria. These findings expand the diversity of Helarchaeota, and suggest that geological products such as hydrocarbons released from thermogenic processes in subduction zones exemplified by the Costa Rica Margin could play an important role in fueling the catabolism of microbial life in the marine deep biosphere. Experimental approaches, such as laboratory incubations complemented with cell visualization, further microscopic observations and transcriptome sequencing [22] are required to confirm these proposed syntrophic interactions.

## Materials and methods

### Study sites and sample collection

Sediments used in this study were collected from Hole U1379B at the Costa Rica margin during the IODP Expedition 334. This site is located on the Caribbean plate shelf into the upper slope, with the water depth of this site is 127 m. Sediments in this Hole were drilled using the advanced piston corer system with the core recovery of 100%. Detail of drilling at this site was provided in [51]. Thorough descriptions of the sediment properties were published elsewhere [27, 30]. Whole-round cores of 10-cm long for microbiology analyses were capped upon drilling using blue plastic caps at both ends, and stored in anoxic plastic bags. Microbiology samples were stored at the Gulf Coast Repository of IODP at −80 °C and then were shipped to the University of Delaware and stored in −80 °C freezer until further analyses.

We in this study focused on the shallow silty clay sediments of 2–9 mbsf from Hole U1379B, which is 20 m south to Hole U1379C where the most comprehensive geochemical profiles at this station were measured. The geochemical profiles of sediment in this interval of U1379C were reported in previous publications: porewater concentrations of sulfate, ammonium, and alkalinity in Expedition 334 Scientists [30], and dissolved manganese in [52]. Procedures for hydrocarbon composition determined by headspace analysis were described in [53], and the data can be found in the IODP Expedition 334 report (http://publications.iodp.org/proceedings/334/334toc.htm).

### DNA extraction, quantitative PCR, and amplicon analysis

DNA for amplicon sequencing was extracted from 0.5 g of sediment using the PowerSoil DNA extraction kit (MOBIO, CA). A parallel extraction blank without adding sample material, in the beginning, was also perform to track the potential contamination introduced during the experimental process. DNA for metagenomic sequencing was extracted from about 5 g of sediment (0.5 g was added into each of the 10 lysis tubes) using the PowerSoil DNA extraction kit (MOBIO, CA). For each sample, DNA extracts from the ten parallel extractions were eluted with 100 µL double-distilled H_2_O into a single 1.5-mL Eppendorf tube and preserved at −20 ^o^C for further analyses.

The bacterial and archaeal 16S rRNA genes were quantified using the primer sets Uni341/Uni519 and Uni515F/Arc908r, respectively, combining with the thermal conditions described in [6]. For the amplicon preparation, a two-step strategy was employed to prepare the amplicons of the V4 region of the 16S rRNA gene with the primer pair Uni519F/806R and thermal cycling condition described elsewhere [6]. Details about the qPCR standard preparation, qPCR reaction chemistry, amplicon sequencing data analysis were presented in the Supplementary Information.

### Metagenome assembly, binning, and genome refinement

Metagenomic libraries (2 × 150 paired-end) were prepared and sequenced on an Illumina NextSeq 500 sequencing platform (Illumina Inc., San Diego, CA, USA). Quality of the reads and presence of adapter sequences were checked using FastQC v.0.11.5 [54] and then trimmed using Trimmomatic v.0.36 [55]. Putative 16S rRNA genes in the trimmed metagenome reads were used to assess the microbial community structure using phyloFlash v3.3 beta 2 [56], in which the short reads were identified and classified against the SILVA 132 release.

The quality-controlled paired-end reads were de novo assembled into contigs using Megahit v.1.1.2 [57] with the k-mer length varying from 27 to 117. Contigs longer than 1000 bp were automatically binned with MaxBin2 v2.2.6 [58] using the default parameters. The quality of the obtained genome bins was assessed using CheckM v.1.0.7 [59] with the option “lineage_wf”, which uses lineage-specific sets of single-copy genes to estimate completeness and contamination and assigns contamination to strain heterogeneity if amino acid identity is >90%. Genome bins of >50% completeness were manually refined using the *R* package *gbtools* [60], based on the GC content, taxonomic assignments, and differential coverages in different samples. To improve the quality of MAGs, metagenome reads of the sample with the highest coverage was detected were mapped onto the MAG contigs using BBmap [61], and the aligned reads were re-assembled using SPAdes v.3.12.0 [62] with the default parameters and minimum contig length of 1000 bp. The resulting scaffolds were visualized and re-binned using *gbtools* [60] as described above. The qualities of the resulting MAGs were checked using the CheckM “lineage_wf” command again. Details were presented in the Supplementary Information.

### *mcrA* gene detection using GraftM

Reads of *mcrA* in the unassembled metagenome sequencing data were identified and classified using GraftM v0.13.1 [32], with the pre-curated *mcrA* package (including HMM profiles and pre-constructed phylogenetic tree) described in ref. [33]. as the reference database (downloaded from https://data.ace.uq.edu.au/public/graftm/7/).

### Genome annotation

Genome annotation was conducted using Prokka v.1.13 [63], eggNOG [64], and BlastKoala [65] using the KEGG database. The functional assignments of genes of interest were also confirmed using BLASTp against the NCBI RefSeq database. The metabolic pathways were reconstructed using KEGG Mapper [66]. The gene synteny of the energy-transfer complex in Helarchaeota genomes was visualized using GeneSpy v1.2 [67], based on the GFF files from Prokka annotation.

For the multi-heme cytochrome detection, the heme-binding sites of a protein, the CXXCH motif, were counted using the python script “cytochrome_stats.py” described in ref. [68] (available at https://github.com/bondlab/scripts) with the amino acid sequences predicted with Prodigal [69] as the input. Proteins with >3 CXXCH motifs were counted as multi-heme cytochromes following the criteria described elsewhere [68, 70].

Average nucleotide identify between different genomes was calculated using FastANI with default parameters [71]. Average amino acid identify (AAI) was calculated using CompareM (https://github.com/dparks1134/CompareM) with the “aai_wf” option, in which the protein-coding sequences predicted by Prodigal [69] were taken as the input to identify orthologous proteins by all-vs-all reciprocal sequence similarity search with Diamond [72]. The average similarity of the orthologous proteins between the two genomes was taken as the pairwise AAI.

### Phylogenetic analysis

For the phylogenomic analysis of Helarchaeota, representative genomes of all major archaeal lineages described in the GTDB database [44] were downloaded and included. For sulfate-reducing bacteria, representative genomes of the candidate bacteria phyla of Desulfobacterota, Desulfobacterota_A, and Desulfuromonadota (phyla per nomenclatures of GTDB; https://gtdb.ecogenomic.org/) were included. The phylogenomic analyses were based on markers consisting of 15 concatenated ribosomal proteins (rpL2, 3, 4, 5, 6, 14, 16, 18, 22, 24, and rpS3, 8, 10, 17, 19) that have been demonstrated to undergo limited lateral gene transfer [73]. These selected proteins, among the conservative single-copy ribosomal proteins included in [74], were identified in Anvi’o v.5.5 [75] using Hidden Markov Model (HMM) profiles, following the procedure outlined at http://merenlab.org/2017/06/07/phylogenomics/. Sequences were aligned individually using MUSCLE [76], and alignment gaps were removed using trimAl [77] with the mode of “automated”. Individual alignments of ribosomal proteins were concatenated. The maximum-likelihood phylogenetic tree was reconstructed based on the concatenated alignment using IQ-TREE v1.6.10 [78] with LG + F + R6 as the best-fit evolutionary model selected by ModelFinder [79] and 1000 ultrafast bootstraps using UFBoot2 [80].

A maximum-likelihood phylogenetic tree based on 16S rRNA genes was also constructed based on an alignment of 16S rRNA gene sequences of the genomes included in the above-mentioned phylogenomic analysis. In addition, amplicon OTUs (287 bp) of Asgard archaea and their close relatives (environmental clones of >1300 bp) identified via BLASTn [81] in the NCBI database were also added. Sequences were aligned using MAFFT-LINSi [82], and putative insertions were manually trimmed in Unipro UGENE [83]. The maximum-likelihood phylogenetic tree was inferred based on the insertion-free alignment using IQ-TREE v1.6.10 [78] with SYM + R5 as the best-fit evolutionary model determined by ModelFinder [79] and 1000 ultrafast bootstrap replicates using UFBoot2 [80].

For the phylogeny of McrA, in addition of Helarchaeota, the genomes of known MCR-bearing archaea genomes were downloaded from NCBI, annotated using Prokka v1.13 [63], and the McrA amino acid sequences were extracted. Phylogenetic analysis was also performed for the McrA sequences in the bulk assemblies of the four CR sediment horizons, in which the McrA sequences were extracted from the Prokka annotation outputs. In both analyses, all sequences were aligned using MAFFT-LINSi [82], trimmed using trimAl [77] with the mode of “automated”, and the maximum likelihood phylogenetic tree was inferred using IQ-TREE v1.6.10 [78] with LG + F + R6 as the best-fit evolutionary model and 1000 fast bootstrap replicates.

For the phylogeny of [NiFe] hydrogenases, reference sequences were mainly extracted from refs. [84, 85]. [NiFe] hydrogenases of the genomes reported in this study were extracted from the Prokka annotations, and used as queries in BLASTp search [81] in the NCBI database to identify their close relatives. All retrieved sequences were aligned using MAFFT-LINSi [82], trimmed using trimAl [77] with the mode of “automated”, and the phylogenetic tree was reconstructed using IQ-TREE v1.6.10 [78], with the procedure described above.

### Genome coverage calculation and the linear correlation

As a proxy of the relative abundance, genome coverages of MAGs in each of the four metagenome-sequenced sediment depths were determined by recruiting reads from the individual metagenome datasets using BBmap [61] with the read identity threshold of 98%. Relative abundances of the Helarchaeota MAGs in the total communities were also calculated as the product of the average coverage and genome size, divided by the total trimmed reads. Linear correlations between the five Desulfobacteria MAGs and the three Helarchaeota MAGs were determined in *R* [86], using Pearson correlation test.

### Thermodynamic calculation

We assessed the feasibility of alkane oxidation processes by calculating the Gibbs free energy for reactions of sulfate-dependent oxidation of ethane, propane, butane as well as methane in the shallow sediments (<10 mbsf) (See Supplementary Table S2 for the chemical equations). Details are presented in the Supplementary Information.

## Supplementary information

Supplementary Information

## Data Availability

All sequencing data used in this study are available in the NCBI Short Reads Archive under the project number PRJNA599172. In particular, the amplicon sequencing data can be accessed through the BioSample numbers SAMN13740702–SAMN13740710. The raw metagenomic sequencing data are available in NCBI under the BioSample numbers SAMN13740741- SAMN13740744. The MAGs described in this study are available in NCBI with the accession numbers JABXJT000000000–JABXKA000000000.
